# Clinical Lectures upon the Mental and Nervous Diseases

**Published:** 1870-01

**Authors:** 

**Affiliations:** Sainte Anne, Paris


### Original Translations.

ARTICLE I.—*Clinical Lectures upon Mental and Nervous Diseases; Alcoholism, Alcohol and Absinthe, Absinthic Epilepsy.* By M. MAGNAN, Sainte Anne, Paris.

(CONTINUED FROM PAGE 696, VOL. XXVI.)

ALCOHOL, in man, as an animal, is incapable, alone, of provoking epilepsy; it causes tremor, but that is all. When, on the other hand, epileptic attacks supervene, you never fail to discover that a different agent from alcohol has provoked them, and this agent is usually absinthe.

Epilepsy is, then, I repeat it, an epiphenomenon, which can not be considered as the highest manifestation of acute alcoholism. Among the patients whom you will examine to-day there will be found a man aged forty-one years, a market porter, who, after repeated excesses with wine and brandy, devoted himself more especially to absinthe. He had had, up to this time, tremors and some hallucinations, when three days ago he was suddenly seized, at the market, with an epileptic attack. He fell senseless immediately; his countenance was distorted, at first very pale, it became violet, the lips were covered with froth, the arms and the legs exhibited convulsive struggles; at the end of a moment the patient aroused himself stupified thoroughly. Some hours afterward he became violent, declared himself pursued by assassins, saw animals around him. He entered yesterday the examining office, his delirium continues, with frightful hallucinations, but one point upon which I wish to direct your attention is the condition of mobility: you will perceive a tremor of the hands, quite considerable, doubtless, but nevertheless not at all exaggerated, less severe even than in a case of acute alcoholism of medium intensity; there has been then no successive progression of motor disturbances terminating in the epileptic attack; this has been superadded, momentarily, to the ordinary phenomena, but without exercising, either before or after, any appreciable influence upon mobility.

I might furnish you with the recital of a large number of analogous observations, collected by M. Bonchereau and by myself, but they would throw no new light upon the question which we are considering. However, I must recall to you an incident which I had the opportunity to observe at Bicêtre, and in which the man, to a certain extent, submitted himself as the subject of experiment, in order to serve as a demonstration of the effect of absinthe.

Cl. . . . (Louis), aged thirty-two years, entered the Bicêtre the 31st of October, 1863. This man, in excellent health, remained sober up to the beginning of 1861, at which date he became a wine merchant. He contracted, at this time, the habit of drinking; he took at first wine and brandy, then a little absinthe.

Alcoholic phenomena soon appeared, he experienced also vertigo especially. During the course of 1863 Cl. . ., to gain strength, made use of the absinthe liquor more freely; the attacks of vertigo became more frequent, and at a few days' interval he sustained two attacks, with sudden loss of consciousness, full, contorted face, convulsions of the arms and legs, bloody froth upon the lips, and biting of the tongue. One of the attacks occurred in church during a funeral ceremony, the other occurred on a stairway; in both cases they occurred unexpectedly.

Delirium, with frightful hallucinations, soon agitated the patient, and necessitated his sequestration at the Bicêtre; he arrived on the 31st of October, 1863, presenting the symptoms of an attack of acute alcoholism. He recovered quite rapidly, and at the end of a month was allowed to leave. On returning to his home he lost no time in resuming his old habits; on the other hand, alcoholic symptoms did not attend him.

Later, in consequence of a fresh abuse of absinthe, he sustained an epileptic attack similar to the preceding.

The patient was brought back the 28th of April, 1864, to the Bicêtre, where he remained, for his recovery, up to the beginning of June.

Discharged for the second time, he abandoned for some time the use of absinthe, but recommenced at the end of a few days to drink wine and brandy. His sleep became disturbed, hallucinations of a painful character manifested themselves; his appetite was lost, streams of pituitary secretion were discharged every morning, and his limbs became tremulous. This condition continued two months, but Cl. . . ., finding himself weaker, had recourse again to his favorite liquor. The absinthe provoked, very soon, new epileptic attacks, C. . . . reëntered the Bicêtre, for the third time, the 5th of December, 1864. At the moment of his entrance he still bore upon his tongue traces of bites, convincing evidence of his last attack. Such was this observation, not to call it experiment. The subject is a young and vigorous man, entirely free up to that time from any alcoholic symptom or any convulsive phenomenon. Commencing with wine and brandy he became alcoholized, then giving himself up to absinthe he became epileptic.

During his first residence at the asylum the symptoms disappeared. Upon leaving he recommenced to drink; alcoholic symptoms at first supervene; he takes absinthe, then comes a new epileptic seizure. Another residence at the hospital, with a cessation of the symptoms. For the third time an excess of wine and brandy induce alcoholism; excess of absinthe, epileptic attacks which are superadded. Again residence at the hospital and sobriety, with cessation of the symptoms.

Can effect and cause be more intimately associated? Can we not, by doubling the dose, induce the effect of both alcohol and absinthe.

Absinthic-epilepsy could not be more clearly demonstrated. How much do these epileptic attacks, always in relation to the determinate agent which produces them, differ in respect to their nature from those which accompany chronic alcoholism? In this last case what happens! A patient presents epileptic or epileptiform symptoms; he enters the hospital, and during the entire duration of his residence we may observe several recurrences of attacks similar to the first under the influence of the most diverse causes, sometimes even without appreciable cause, absolutely as in patients suffering with certain cerebral lesions.

Chronic alcoholism exists indeed under analogous conditions; his cerebro-spinal system has sustained profound and persistent alterations. The prime cause exists altogether in the state of the organism, conditions very opposite to those of poisoning by absinthe, in which the sole cause is the poison.

What is the progress of acute alcoholism?

It would be difficult to form a just idea of the gravity of acute alcoholism from the statistics of the majority of physicians, and more particularly from English and American statistics.

Such wide variations occur in these documents that all comparison is impossible. Hence we believe it more useful, and of more practical interest to confine our investigations to observations collected at Paris, which enable us to form a more exact appreciation of all the elements of the problem.

In 1852 M. Delasiauve,* vividly impressed with the severity of certain cases of *delirium tremens*, published upon this subject an excellent treatise, in which may be found an accurate table of subacute *delirium tremens.*

Although the observations comprise but a small number of facts selected amongst the most important, there will be found, nevertheless, the proof of a very considerable mortality.

We shall draw, in the thesis of Doctor Contesse†, numerous documents, and so much the more valuable that they can in all points be compared to those which we shall obtain here. Among 5,238 patients suffering from various forms of delirium, entered at Bicêtre, from the 1st of August, 1855, to the 1st of April, 1862, M. Contesse found 1,000 alcoholic subjects, amongst whom there were 30 cases of death in the 34 first days after their arrival at the asylum; but the author, comprehending readily that this duration of 34 days would become unreliable by reason of its length, has added a table indicating the *deaths*, day by day, starting from the date of entrance.

In this manner we can, by embracing only the first five days, recognize the figure which reasonably indicates acute alcoholism.

After the 5th day the subjects of acute alcoholism do not die, properly speaking, of intoxication, *per se*, but rather from some of its complications.

Of the 30 deaths we find 21 during the first 5 days; there remains then but nine of them to be apportioned among the 29 days remaining of the 34.

**D'une forme grave de* delirium tremens, Delasiauve, *Revue Medicale et etragère*, 30 Avril, 1852.

†*Etude sur l'alcoolisme et sur l'étiologic de la paralysie générale*, Contesse, 1862.

These results, to which I particularly direct your attention, indicate sufficiently, by themselves, the existence of danger, especially in the acute period, that is to say, at the moment when the entire economy is under the pressure of the toxic impregnation. But among the 1,000 patients the acute and the chronic are indistinctly embraced. It is indispensable, nevertheless, to separate them, for the 21 deaths during the 5 first days, evidently refer only to cases of acute alcoholism.

We have been able to determine this relation by assuming, as a basis, our own results, which I shall submit to you, and we think we can show that, of the 1,000 alcoholic cases, about 260 were the subjects of acute alcoholism, which would give a proportion of eight deaths in a hundred.

You know, gentlemen, why I compare, without the least restriction, the statistical results of the Bicêtre with those of the Examining Office; it is because we have to deal with analogous elements.

Our patients of the Examining Office are different, for the greater part, from the patients who formerly were brought to Bicêtre; hence we find ourselves in identical conditions. The Parisian drunkards of 1862 do not differ, as I well know, from the Parisian drunkards of 1867.

Since the organization of the insane department of the Seine, 1st of May, 1867, up to this time, end of April, 1869, M. Buchereau and I have counted, among 4,866 entries, 231 cases of acute alcoholism, and 662 of chronic or sub-acute alcoholism, in all 893, out of which number we have lost, during the first 5 days, 3 patients only; more exactly, and omitting the chronic cases, 3 deaths in 231 cases of acute alcoholism, which gives 1.30 per cent. at the Examining Office, in place of 8 per cent., which was obtained at Bicêtre, I might even say that we obtained at Bicêtre; for, you know, the patients arrive generally in the afternoon, and the first treatment is given by the internes (resident students). The patients, then, who died on the first day were treated by us. The results obtained by M. Bouchereau are almost analogous to those of M. Contesse and to mine. How then can we explain this difference between 8 per cent. at Bicêtre and 1.30 per cent. at the Examining Office, with the same patients, the same physicians? I may also add that our pharmaceutical treatment at the Examining Office scarcely differs from that which we prescribe at Bicêtre. You perceive, gentlemen, that there is an X, an unknown quantity, and of this unknown quantity we shall now speak.

When a case of acute alcoholism arrived at Bicêtre, what was done?

He was perhaps violent, dangerous to himself, dangerous to the attendants. The first indication to be fulfilled was to protect him against himself, and to prevent him from doing harm to others.

The straight waistcoat was the remedy. This apparatus carefully applied had the effect to exercise a certain restraint over the thoracic movements, especially towards the base, but there the danger was but slight.

The subject of acute alcoholism, in spite of the waistcoat, continues his violence, strikes all around him, wounds himself and necessitates other precautions.

The bed is all ready to receive him. He is placed so much the more readily, that these patients, manifesting always a certain febrile condition, it seems necessary to place them in bed.

The subject of acute alcoholism remains in bed solely under one condition, that he be secured; it thus becomes necessary to fix him there.

To be tied to the bed, is death to the patient. You will understand this.

The patient is extended upon the bed; the straight waistcoat is laced, the arms, crossed in front, are secured by the aid of sleeves on each side of the thorax, and are solidly applied to the chest. This is an excellent means to render the lower part of the thorax immovable, to prevent the play of all the false ribs, and of two or three of the last ribs.

At the upper part of the waistcoat are two rings corresponding to the sub-clavicular region; bands passing through these rings are fixed below and behind at the upper extremity of the bed, and in order that the patient may not slip, that his head may rest easy, the pillow is placed between the bands which pass under the head, and the head is applied to it in such a manner that the whole anterior and superior part of the waistcoat remains tightly strained across the corresponding part of the thorax, which it fixes immovably.

As soon as the chest is thus clamped above and below, the diaphragm is set in motion, and abdomen respiration supplies, up to a certain point, the imperfection of thoracic respiration.

But the patient having his legs free, feeling ill at ease, makes incessant efforts to detach himself from the bed. Naturally this must be prevented. The legs are extended, brought together, and the feet, encircled with shackles, are secured to the extremity of the bed. The result of this is tension of the muscles of the abdominal walls, which especially hinder the efforts of the diaphragm. This is not all. Large folded sheets, placed across upon the belly and limbs, are secured to each side of the bed.

Thus garroted, the patient at first cries vociferously. His countenance becomes purple, his eyes injected; the swollen jugular can scarcely empty themselves; the whole cephalic extremity becomes congested; the countenance becomes puffed and glistening, the neck swollen, sometimes bulging out like a cushion above the rigid ring of the waistcoat, which strangles him. In a state of inexpressible anguish, the miserable patient struggles, and makes superhuman efforts up to that point, where, exhausted, in a state of semi-asphyxia, and always under the active influence of the poison, he falls into a condition of complete resolution.

Death sometimes occurs very suddenly; at other times the face becomes livid, the body is covered with a viscous cold sweat. Involuntary evacuations occur; the pulse is small, tremulous, compressible, with subsaltus tendinum, slight thrilling movements in all the muscles, by degrees respiration is impeded, all the functions are arrested insensibly, the patient is quietly extinguished.

Such are the severe cases. When the patient resists, quite frequently he falls into a semi-comatose sleep; the state of resolution in which he lies induces a relaxation of the whole organism; respiration and circulation, although impeded, are gradually reëstablished. Profuse sweats cover the body, and after a variable number of hours, the patient awakens, crushed, sometimes tranquil, at other times preserving a certain degree of excitement, betraying itself by incoherent words, by feeble cries, and by efforts, which his exhaustion renders powerless.

Let us examine now how we may, at the Examining Bureau, fulfill this first indication, viz., to protect the patient against himself and to prevent deeds of violence to those around him.

I speak to you so much the more freely because the original merit belongs entirely to the administration which, at its first organization, was sufficiently intelligent to place in our hands the means of action necessary to secure good results.

As the alcoholic patient is in a state of extreme agitation we place him in swaddling clothes, which we have substituted for the straight waistcoat. This swathe of cloth, in winter, and of linen in summer, adapts itself exactly to the surface of the body, without exercising pressure upon any point. The arms remain pendant, and are retained in this position by the aid of lateral pockets, into which the hands enter; these are retained by the sleeve itself, fastened in its turn to the portion of the swathe which serves for pantaloons. We employ for women this same form of swathe by adding to it a petticoat.

I will exhibit to you an acute alcoholic subject, excited, and you will see, thanks to the position of the arms, maintained along the sides of the swathe, to be the reverse of what happens with the waistcoat. The greater the agitation the more easily is the respiration accomplished. In some cases the swathe and the presence of a keeper are not sufficient to prevent accidents.

We then place the patient in a large, well ventilated cell, having its wall covered with a thick layer of hair, kept in position by strong sail-cloth, stretched very tight and well varnished upon its exterior surface. Upon the floor are placed two palliasses, placed one upon the other, covering the whole floor, the first extending in a single piece over the whole surface. The second, composed of several pieces, which may be changed as soon as they become soiled.

In the corner is placed a caoutchouc vase. On the floor is a mattress covered with clothes and coverlets, with a bolster and pillow.

The keeper remains before the door, and through a little loophole observes what passes.

This fulfills the first indication; the patient may execute the most disorderly movements, or give way to the most active violence; there is nothing to be feared.

All the functions are regularly performed; and the greater his activity the more rapidly will the poison be eliminated by the perspiration and respiration.

This leads me to speak of the second indication, to facilitate the elimination of the poison.

At this point we avail ourselves of the knowledge acquired in our physiological studies.

We know that alcohol is not transformed, that it escapes in the state of alcohol, and that the danger will pass away so much the more quickly as the elimination is more rapid and more complete.

Thirst is generally acute; it should be satisfied, by inundating, as it were, the alcoholic patient with aqueous drinks, diluent or slightly aperient. Citric acid, lemonade, tisane of couch grass (chiendent), slightly nitrous, barley water, with a little cream of tartar, are usually used. Under these circumstances, if no complication supervenes, the poison is speedily expelled by the lungs, the kidneys and the skin.

But at this period of effervescence, of nervous hyperæsthesia, succeeds a last phase, of totally different physiognomy, viz., collapse, nervous exhaustion; a state of extreme prostration, from which the patient is extricated with the greatest difficulty.

This condition, which demands consideration, furnishes matter for a third indication,

To build up the strength.

We have recourse to tonics, more particularly to cinchona, and by preference to the extract, which we use largely.

We administer broths, soups, meat essence, and even meat itself, as soon as it can be borne.

And lastly, it is sometimes good to give a little wine.

To epitomize. 1st. Protect the patient; 2nd. Eliminate the poison; 3rd. Support the strength. Such are the symptoms which present themselves ordinarily in acute alcoholism.

Among the simple means of fulfilling these indications, of which I have just spoken to you, you have doubtless remarked that there has been no suggestion of opium, nor of digitalis, nor of chloroform, nor of alcoholic diet, nor of blood-letting, etc.

If the unfortunate results obtained with these different medicaments did not suffice to induce their rejection, what we know at this date, of acute alcoholism, should compel us to proscribe them, absolutely, except with very rare exceptions, which should, of themselves, originate in the presence of complications altogether special.

